# Heat Sterilization Effects on Polymeric, FDM-Optimized Orthopedic Cutting Guide for Surgical Procedures

**DOI:** 10.3390/jfb12040063

**Published:** 2021-11-19

**Authors:** Leonardo Frizziero, Gian Maria Santi, Christian Leon-Cardenas, Patrich Ferretti, Merve Sali, Francesco Gianese, Nicola Crescentini, Giampiero Donnici, Alfredo Liverani, Giovanni Trisolino, Paola Zarantonello, Stefano Stallone, Giovanni Luigi Di Gennaro

**Affiliations:** 1 Department of Industrial Engineering, Alma Mater Studiorum University of Bologna, 40136 Bologna, Italy; gianmaria.santi2@unibo.it (G.M.S.); patrich.ferretti2@unibo.it (P.F.); merve.sali2@unibo.it (M.S.); francesco.gianese@studio.unibo.it (F.G.); nicola.crescentini@studio.unibo.it (N.C.); giampiero.donnici@unibo.it (G.D.); alfredo.liverani@unibo.it (A.L.); 2 Pediatric Orthopaedics and Traumatology, IRCCS–IOR—Rizzoli Orthopaedic Institute, 40136 Bologna, Italy; giovanni.trisolino@ior.it (G.T.); paola.zarantonello@ior.it (P.Z.); stefano.stallone@ior.it (S.S.); giovanniluigi.digennaro@ior.it (G.L.D.G.)

**Keywords:** HTPLA, nylon FDM, FDM, 3D engineering, sterilization, cutting guide, preoperative planning

## Abstract

Improvements in software for image analysis have enabled advances in both medical and engineering industries, including the use of medical analysis tools to recreate internal parts of the human body accurately. A research analysis found that FDM-sourced elements have shown viability for a customized and reliable approach in the orthopedics field. Three-dimensional printing has allowed enhanced accuracy of preoperative planning, leading to reduced surgery times, fewer unnecessary tissue perforations, and fewer healing complications. Furthermore, using custom tools chosen for each procedure has shown the best results. Bone correction-related surgeries require customized cutting guides for a greater outcome. This study aims to assess the biopolymer-based tools for surgical operations and their ability to sustain a regular heat-sterilization cycle without compromising the geometry and fit characteristics for a proper procedure. To achieve this, a DICOM and FDM methodology is proposed for fast prototyping of the cutting guide by means of 3D engineering. A sterilization test was performed on HTPLA, PLA, and nylon polymers. As a result, the unique characteristics within the regular autoclave sterilization process allowed regular supplied PLA to show there were no significant deformations, whilst annealed HTPLA proved this material’s capability of sustaining repeated heat cycles due to its crystallization properties. Both of these proved that the sterilization procedures do not compromise the reliability of the part, nor the safety of the procedure. Therefore, prototypes made with a similar process as this proposal could be safely used in actual surgery practices, while nylon performed poorly because of its hygroscopic properties.

## 1. Introduction

Recent developments of 3D printing processes have been applied to the biomedical engineering field in areas such as tissue engineering and generative medicine [1]. Furthermore, several studies have different sorts of applications of 3D-printed organs and tissues [2]. Meanwhile, research on 3D medical procedure design included a hands-on process for the surgical operations related to the manufacturing of organs and tissues in case the doctor does not have a direct connection with the organ development area [3]. Currently, in the medical field, real-life training is becoming more challenging, as traditionally accepted methods, such as direct supervision or corpse training, have shown to be difficult because of the high number of students [4]. Radiological solutions such as computed tomography (CT) can show body layers by means of their density to create cross-sectional images of a particular area of the human body [5,6]. Furthermore, anatomically accurate physical models evolved from CT and magnetic resonance imaging (MRI) data by using three-dimensional (3D) printing technology [7] showed variations in a later study [8]. Subsequently, a patients’ pathology could be reproduced using CAD models and 3D printing technology, and the creation of tissue structure models has also been unveiled [9].

**Figure 1:**
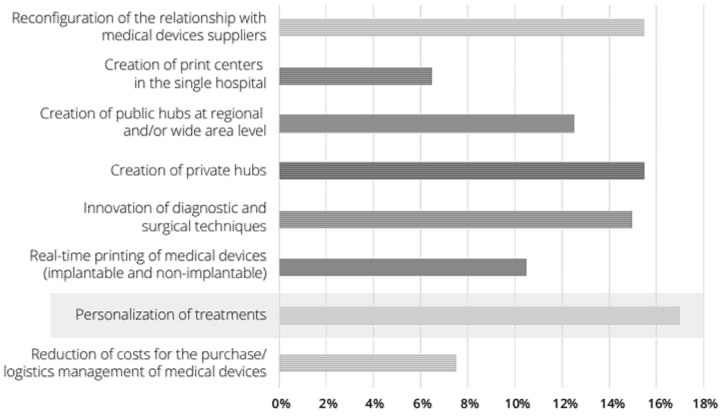
Three-dimensional printing opportunities for hospitals.

Moreover, augmented reality-based technology by means of the computer-aided surgical simulation (CASS) methodology proved to increase the final operation outcome [10], thus decreasing difficulties and subsequent issues, which could also lower costs in the medium term for all parties involved. Additionally, it showed an increase in apprised consent and helped to have clearer communication between doctors and patients; with a 3D model, surgeons can easily convey medical information and chosen treatments for the case, building a deeper bond of trust with the patients [11]. Additionally, 3D design and part construction is applied in the medical area nowadays by developing patient-customized body models to allow surgeons to plan the procedure, produce in-house anatomical representations for medical training, and print surgical tools, prostheses, and implants as well [12]. Although 3D printing applied in the medical area could differ from treatment preparation, surgical guide design, teaching prototypes, and educational tools are made to create scaffolds for tissue engineering and printing of tissues and other organs [13]. The hospitals recognize great potential in different fields and, in particular, in the personalization of treatments (i.e., tailormade prostheses) and in the creation of public hubs at the regional or supra company level (Figure 1) [14]. Nevertheless, by applying rigorous regulation on some elements (e.g., the use of bioprinting practices, prostheses, artificial organs, etc.), 3D printing would emerge as a major technique for instruction and groundwork of complex surgical procedures [14]. Further discoveries propose to appraise the application of 3D models in other areas of surgical practices, for example, training for patients prior to undergoing operations, using 3D models as a preoperative [15] and intraoperative tools, and integrating such models into patient-oriented procedures. This would signify the adoption of this technology, encouraging more medical professionals to apply these models in their practices [16]. Subsequently, the study of Sampogna et al. [17] performed an assessment of three tactics on 20 surgeons, showing that most surgeons quizzed scored their surgery quality and efficacy as good enough.

Furthermore, several surgical strategies exist to ensure accuracy in bone corrective procedures, and tool customization is vital for guaranteeing the best results in these surgical procedures, for instance, the custom-made, high-resistant, machined plates proposed by Brunso et al., which are made in titanium with CAD-CAE [18]. Nevertheless, proper surgical planning is crucial for achieving a perfect correction, and thus, its location and size are vital points to consider when performing an accurate rectification, avoiding new distortions [19]. In order to improve the precision of bone axis alterations, these procedures have recently been used to reach the desired outcome [20]. Therefore, easy-to-handle tools, such as customized cutting guides, could help surgeons to increase the precision of a surgical procedure [21]. Cutting guides are easy to place and generally ensure unequivocal positioning [22]; custom cutting guides have recently been introduced as a more accurate and efficient methodology for orthopedic surgery [23]. Additionally, modern software and 3D printing tools and typologies could help surgeons develop custom cutting guides and wedge spacers, leading to results with higher accuracy in the osteotomy. Surgeons who develop careful preoperative planning and exploit the use of 3D printed guides would benefit from advantages such as less intraoperative time, a decrease in fluoroscopic time, and overall cost, as well as requiring less experienced surgeons for the procedure [24,25].

Subsequently, FDM-sourced parts would need to be sterilized before entering an operation room, and this process could affect the materials exposed to the procedure. Studies in the literature reported that FDM technology is being used to produce custom-made surgical guides [26] and instruments such as handles [27], scalpels and retractors [28], as well as forceps, clamps [29], and scissors [30]. Therefore, it is imperative to understand how the mechanical properties of 3D-printed medical tools, or implants used as instruments, are modified when prepared and then sterilized a few times in a day [31]. Surgical instruments are kept on shelves in autoclave containers and subjected to sterilization cycles before every use [32]. Different methods to sterilize elements exist in hospitals, the most available of which include steam heat sterilization, as well as ethylene oxide (EtO) and gas plasma [33]. The effect of heat sterilization processes on the mechanical behavior of a given material was also assessed to find out the causes of failure [34]. Regulatory bodies in the medical manufacturing industry do not address 3D-printed plastic products [35]. In addition, when using chemical sterilization methods, defects in 3D-printed products would inadvertently result in sterilant penetration from the subsurface to the outer surface [36]. Additionally, rough exterior surfaces could lead to increased surface degradation [37]. Nevertheless, as the popularity of 3D printing in the medical sector increases, common sterilization practices must be analyzed on such manufactured elements [38]. In order to apply heat during sterilization procedures, four methods are popular for medical gear—namely, using an autoclave, EtO, hydrogen peroxide gas plasma, or gamma radiation. This is provided by the CDC’s Guideline for Disinfection and Sterilization in Healthcare Facilities (2008) [39]. As heat sterilization could harm tools, particularly those with a lower tolerance to heat and those repeatedly exposed to it, leaving wet surfaces and risk for burn injuries by handling them [39], as this process heats up to 135 °C under pressure. Higher temperatures would alter the internal print structure, since materials such as PLA have a lower glass transition temperature (Tg) [13]. Furthermore, steam is widely known to sterilize elements such as most metals, glass, and many heat-stable polymeric materials (e.g., polytetrafluoroethylene (PTFE), polypropylene, nylons, polycarbonate, acetal, and even polysulfones), as well as other products such as liquids, fabrics, celluloses, pharmaceuticals, drugs, and medical devices. However, there are materials from the latter category that cannot be treated by other methods [40]. Therefore, sterilization of polymers could be obtained by limited procedures, considering that dry-heat or steam sterilization may lower polymer characteristics and melt it. ETO would leave toxic remains, further oxidizing agents, and hydrogen peroxide can oxidize and harm materials’ external composition. Additionally, the molecular structure of many polymers could be modified by means of radiation (e.g., through cross-linking, scission) that would trigger odor and color fading, embrittling and damaging of some materials, and affecting bonding strength of materials and changing their stability over time [41]. Plastic tools should be assessed after heat sterilization, as their melting temperature and chemical reactions created during thermal degradation could cause changes in their optical and physical properties, compared with the initial values [41]. Moreover, other known sterilization procedures have also proved problematic when using polymeric materials, as ETO could lead to a change in the polymer structure, leading to lower molecular weight values and surface erosion by toxic residues; thus, ETO is not advised for PLA or PETG [42]. 

Ultimately, the main aim of this study was to gain a better understanding of the behavior of three different polymeric materials used to build an orthopedic surgical tool that must endure a regular heat steam sterilization process. This assessment would provide a key conclusion regarding the safety of the application of 3D-printed elements with biopolymeric-sourced filaments in the manufacture of surgery tooling, designed using augmented reality methodologies that would help lower the risk of surgical procedures, with higher efficacy. The applied methodology was rated for its effectiveness in producing a cutting guide with accuracy, reaching required tolerances for its use in the operating room.

## 2. Materials and Methods

### 2.1. Materials

Three different materials were tested in this study, all of which were proven to reach acceptable quality levels from the manufacturing point of view, as well as the possibility of being used in medical applications for their biostability characteristics.

Nylon filament is more flexible than other polymers such as ABS and PLA but has a higher tendency to warpage and will absorb water if not properly stored. This material is more difficult to print than conventional filaments but can produce structurally solid yet flexible models that are useful in the construction of medical devices such as dental instruments [43]. Additionally, this material has been studied by various researchers because of its toughness, elasticity, and wear resistance [44,45,46,47,48,49,50,51,52,53,54]. FDM nylon processing was explored for feedstock materials as a matrix in composite systems, as well as its biocompatibility capacity in the production of scaffolds for tissue regeneration [44,55,56]. 

Polylactic acid (PLA) is a biodegradable material, as it can be made from renewable resources, and has good properties at a low cost, as compared with other traditional biodegradable polymers used in medical applications [57]. It has been widely used for biomedical purposes such as sutures [58], soft-tissue implants, drug delivery devices and tissue-support scaffolds [59], as well as nails, pins, and anchors [60]; further studies were found in the literature on the use of this material in craniofacial augmentations in plastic surgery [61], as well as stents ([62,63,64,65]), screws [66], and spinal cages [67]. PLA is adaptable to different loading environments depending on the characteristics of each application, and its popularity among bioplastics has increased in the past five years due to ongoing applications in a wide range of areas, including for medical purposes [68].

Although PLA has higher print qualities and mechanical characteristics than most other plastic filaments (with the exception of some polycarbonate, nylon, or composite blends [69,70], and acrylonitrile butadiene styrene (ABS) [71]), this polymer has a low fusion temperature that is only acceptable to keep print shape at mild temperatures.

**Figure 2:**
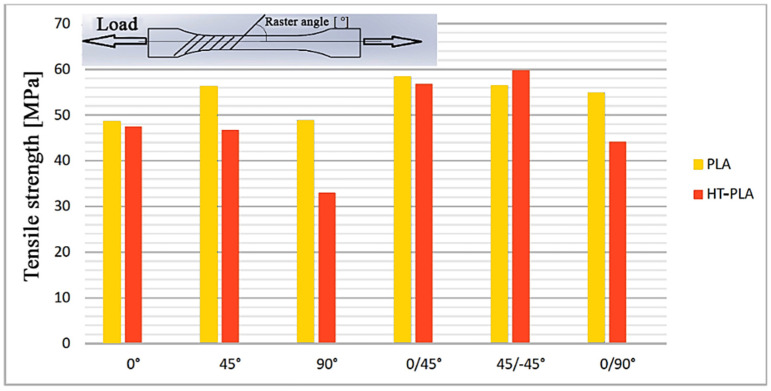
Tensile strength of HTPLA and PLA at different raster angles.

Finally, heat treatable polylactic acid (HTPLA), which is a PLA filament with a crystallization compound, enables the polymer to stabilize toward higher temperatures, and further mechanical improvements could be achieved as well. However, the parameters of 3D printing processes using HTPLA are little studied in the literature. The tensile strength values of HTPLA are shown in Figure 2; research from Hanon et al. concluded that the yield limit of HTPLA has almost entirely disappeared, in contrast to stock PLA [72]. Mechanical properties of used filaments obtained from manufacturers are given in Table 1. 

### 2.2. Methodology

#### 2.2.1. From TAC Image to the Drawing Board, to the 3D Printer

**Figure 3:**
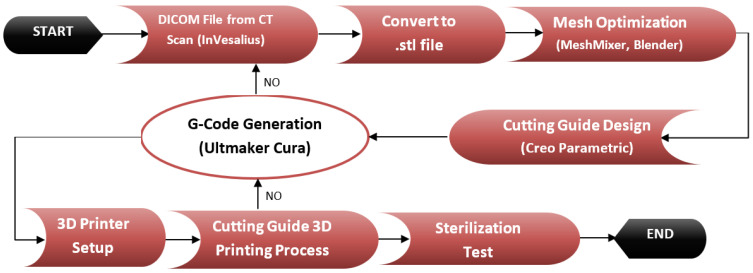
Proposed methodology.

**Figure 4:**
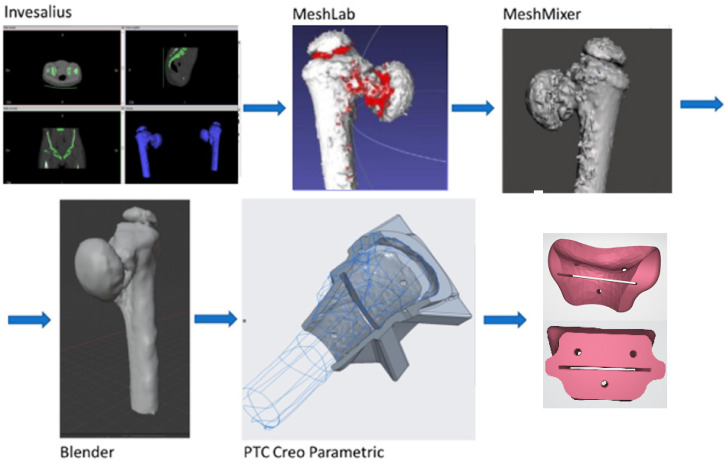
Process for obtaining the bone-cutting guide.

The complete methodology used in this project is described in Figure 3. The process started with obtaining information from a CAT image of the lowermost part of the human abdomen that was analyzed by means of the freeware software InVesalius v3.1, according to which a preliminary 3D mesh of the desired bone was obtained (Figure 4); this mesh was then exported to stereolithography (STL) format. After the mesh was cleaned and optimized for edition and handling by means of different freeware software such as MeshLab v2020.07 (ISTI - CNR, Pisa, Italy), Mesh Mixer v3.5 (Autodesk Inc., San Rafael, CA, USA), and finally with Blender v2.9 (Blender Foundation, Amsterdam, The Netherlands), which allowed a better printing quality and less printing time. The optimized 3D bone model in STL format was used for the design of a cutting guide with the use of the student version of Creo Parametric v7.0 (PTC Inc., Boston, MA, USA) Software. FDM printing coding was possible with the use of a freeware version of Ultimaker Cura v10 (Ultimaker, Amsterdam, The Netherlands) as well, in which the mesh was divided into layers according to the printer and nozzle parameters for each tested material (Table 2), creating the tool path on the build platform. This process allowed the support structure to be formed and scaled accordingly. The final G-code file was obtained after several print simulations in the software in which printing parameters were monitored for ensuring the best-printed part quality and detail overall. The printing machine was run after setting the parameters and inserting the filament into the extruder. Each material layer was laid as intended until the cutting guide with its support structure was completed.

**Figure 5:**
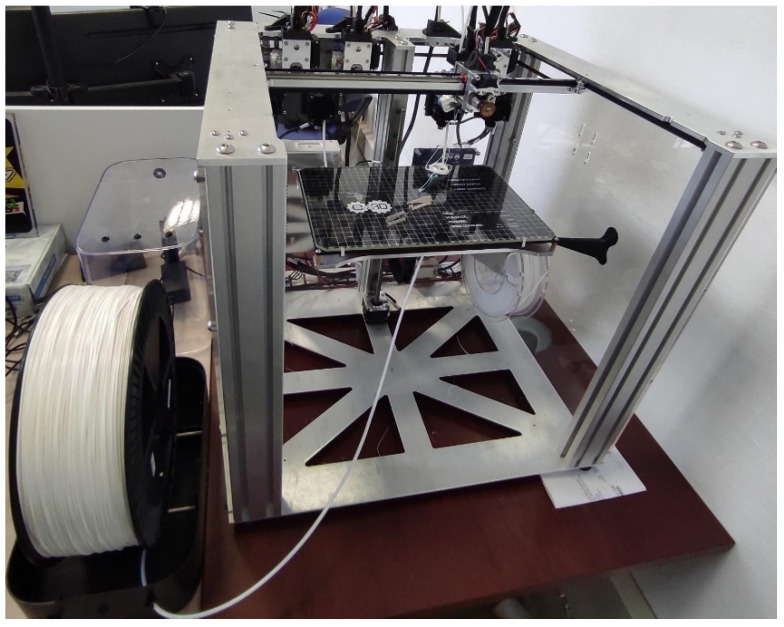
E-3D ToolChanger 3D Printer.

**Figure 6:**
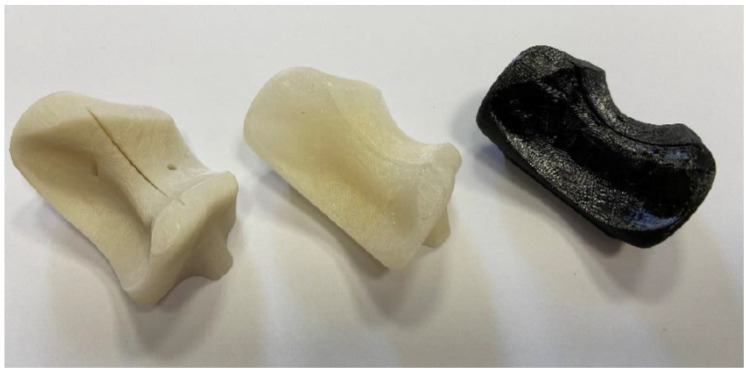
Cutting guides printed by FDM in PLA (**left**), HTPLA (**center**), and nylon (**right**).

Each printed layer was 0.2 mm thick for all cases. The customized 3D printer (Figure 5) used for the project was monitored to ensure no errors were performed. Once finished, the printed object was removed from the platform and cooled down at room temperature before handling. Printing parameters used for the tested materials (Table 3) were obtained using a parameter optimization procedure presented in previous research by Ferreti et al. [73] in which the cutting guides obtained for testing ensured internal material isotropy (Figure 6). All specimens were printed with E-3D ToolChanger 3D Printer (E3D-Online, Chalgrove, UK) (Figure 5); all technical details about the printer are listed in Table 2.

#### 2.2.2. FDM Process Parameters

The mechanical properties of 3D-printed polymeric components were influenced by various factors that can be divided into “constant” and “controllable” factors. The analysis of the manufacturing procedure was performed by following the optimization methodology first introduced by Ferretti et al. [73]. Controllable factors include, for example, the thickness of the layer and the raster angle, while constant factors are precisely the parameters that cannot be changed, such as the nozzle diameter. All the specimens were printed at the same speed, according to the methodology for optimized parameters. Thus, particular consideration was given to certain print parameters that might influence the final outcome. These parameters are as follows:

1. Extrusion multiplier: This is a parameter that determines the quantity of material to add to the standard value of the slicing software and is important since it allows the reduction in defects such as the deposition of material between lines.
2. Extrusion width: as a property indicating the distance between centers of two continuous filaments, this parameter allows the online width to be controlled, but it is impossible to have this value for a single line.
3. Outline overlap: This parameter increases infill quality. A value of 30% showed the best outcome.

#### 2.2.3. Heat Treatment before Sterilization Test

This post-printing process is very often adopted with polymeric materials; it offers the possibility of improving the mechanical characteristics by relaxing the internal tensions to finally achieve more stability at temperatures higher than the polymer’s glass transition temperature (Tg). Annealing is one of the best ways to relieve internal material tensions, reduce imperfections (improves interlayer adhesion), as well as stability and geometry of the final sample. Therefore, an additional cutting guide with HTPLA was created to undergo this process to gain an understanding of a second heat cycle for the printed element. Annealing cycle characteristics were taken both from the filament supplier recommended value and from various tests based on the results submitted by Kočí [74] at an annealing temperature of 130 °C, as higher temperatures would guarantee better interlayer bonding, thus increasing its isotropy, and by iterating at various cycle times until reaching material change across its whole section. The best-obtained results were gathered at 30 min of exposure. The piece was cooled down at room temperature; thus, its structure remained unchanged. 

#### 2.2.4. Autoclave Temperature Analysis

It has been shown in previous research findings that FDM could be used for medical implant manufacturing using bioimplantable materials [75]; however, a study on dental surgical guides established that medical devices using this material are in direct contact with unprotected tissue and bone, as well as blood and other body fluids during the procedure. This creates a potential risk of pathogen contamination that would cause infections. The cutting guide is considered a vital element to sterilize using steam heat, according to the American Dental Association [76,77].

Regulation of medical procedures made the sterilization of printed prototypes before their use in operating rooms mandatory. This process should be reliable, functional, and safe for the devices [78]. Trials for sterilization on dental implants, using autoclave-sourced vapor at 121 °C for 20 min or 134 °C for 4 min [79], although it could also be achieved with high-level disinfectants such as 70% diluted ethyl alcohol for 40 min, are also issued [80]. This process is somewhat affected by inorganic or organic soils and would penetrate medical packaging and device lumens easily [39].

Moreover, steam sterilization follows the EN 285: 2015 Standard (Table 4), recognized as the European standard for the hospital sector, which provides guidelines on three parameters most crucial during operations: temperature, pressure, and exposure time. The following table summarizes three possibilities of sterilization treatment approved by this legislation and frequently used by hospitals [81].

A sterilization process with a long cycle time was used in the autoclave. A comparison was made between PLA, HTPLA, annealed HTPLA, and nylon cutting guides, since it is known that annealing treatment brings important benefits regarding the materials’ thermal stability. Figure 6 shows the cutting guides that were sterilized without having undergone a previous heat treatment to understand how they behaved in the autoclave. The cutting guides at the autoclave followed a very precise cycle, imposed by the Rizzoli hospital, using a Cisa 6410 model autoclave (Lucca, Italy). The sterilization process followed various steps: conditioning, heating, washing, and drying. The actual cycle was much longer than that shown in the previous table, with a duration of about 50 min total and maximum temperatures, which ranged between 134.7 and 134.9 °C.

**Figure 7:**
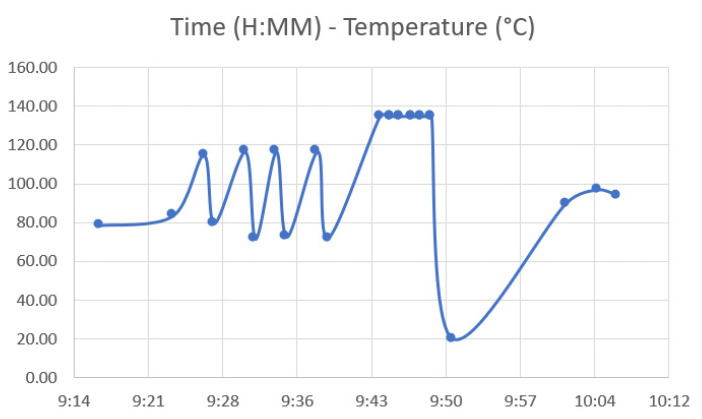
Time—Temperature for autoclaving cycle.

Figure 7 shows the performance of the autoclave cycle as a function of time and temperature, in which the temperature at working conditions is clearly visible at any time point. Additionally, Table 5 shows the entire work cycle parameters considered: time, temperature, and pressure.

### 2.3. Dimensional Assessment after Sterilization Procedure

**Figure 8:**
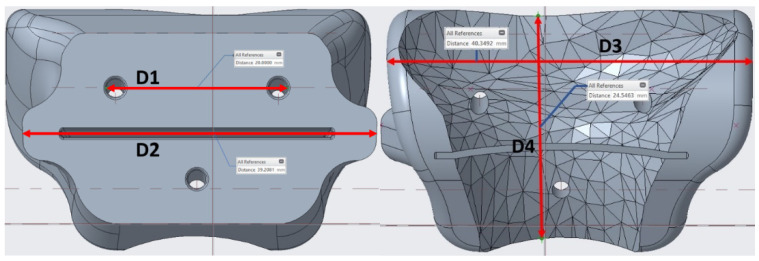
Reference distances for temperature analysis.

The heat treatment response in the guides differed for each material; after testing the product’s fitness on the bone following previous sterilization, a geometrical modification test had to be performed. To this end, four main distances that were the most decisive parameters to guarantee a desired fit on the bone were taken from the product, as shown in Figure 8. Thereafter, a comparison was made between the measurements obtained from Creo software for specific points of the guide and the pieces found after printing; post-autoclave measurements were taken as well.

By identifying these four dimensions on the guides, it could be understood how the geometry has changed during the sterilization process.

## 3. Results

### 3.1. Quality Assessment of the Product after Sterilization

**Figure 9:**
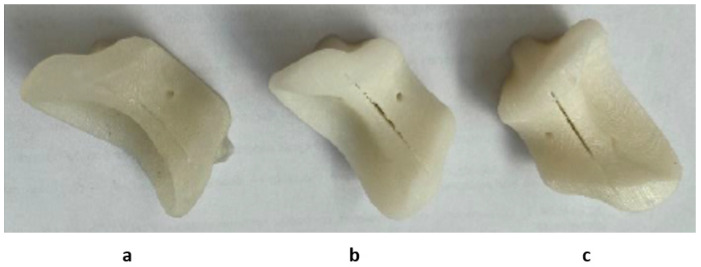
HTPLA cutting guides in 3 different conditions: (**a**) after printing, (**b**) annealed, and (**c**) annealed and sterilized.

**Figure 10:**
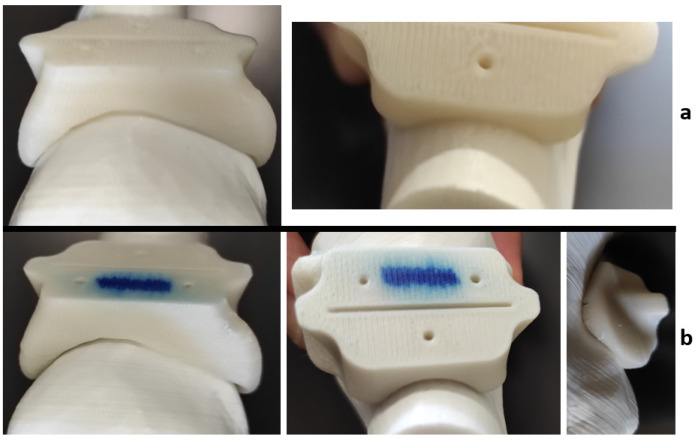
PLA cutting guide bone fit test comparison: (**a**) before Sterilization and (**b**) after sterilization.

**Figure 11:**
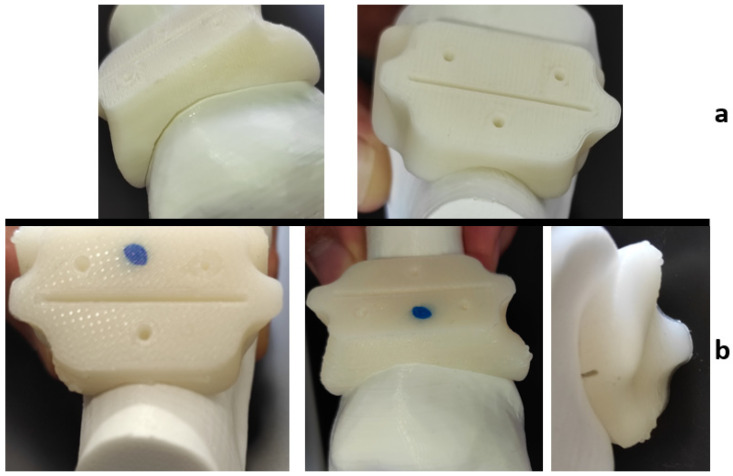
HTPLA-annealed cutting guide bone fit test comparison: (**a**) before sterilization and (**b**) after sterilization.

It is expected that the materials undergo slight visible changes due to the heat processes discussed in this study; Figure 9 shows the differences between three identical HTPLA guides under the different conditions of this research. However, all cutting guides tested in this study were subject to a fit test on the corresponding bone (also obtained by FDM printing) after sterilization. The PLA-filled element in Figure 10 shows that changes in the product after sterilization were minimal to such an extent that are almost undetectable with the human eye, only noticeable as a small variance in the lowermost bone cavity of the element. Conversely, the HTPLA-annealed cutting guide in Figure 11 shows aesthetic differences after both heating cycles, as internal material crystallization due to its inherent characteristic created a glossy surface; nevertheless, the product showed little geometrical and fit modifications, and therefore, its fit accuracy was not compromised, showing an even better fit than PLA.

**Figure 12:**
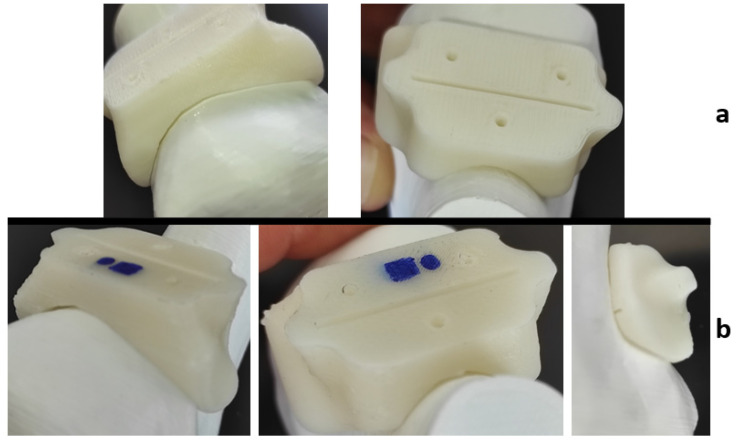
HTPLA cutting guide bone fit test comparison: (**a**) before sterilization and (**b**) after sterilization.

**Figure 13:**
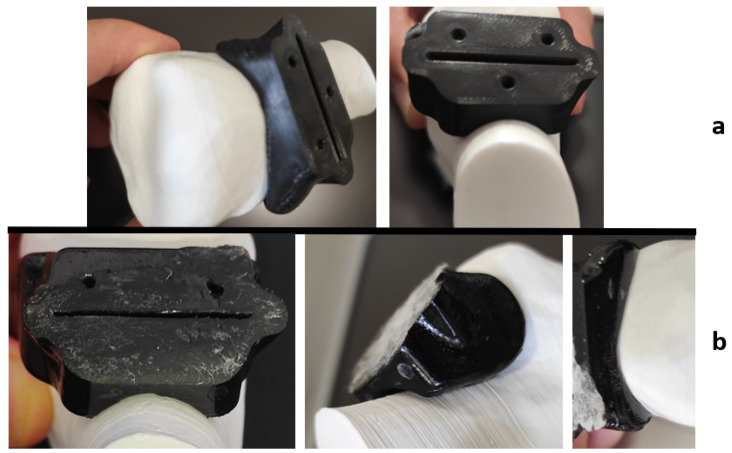
Nylon cutting guide bone fit test comparison: (**a**) before sterilization and (**b**) after sterilization.

However, visible inaccuracies were observed in the cutting guide made of HTPLA, which, without passing through a previous heat cycle before the sterilization, as seen in Figure 12, showed few but noticeable polymer-dried drops and outer surface roughness (Figure 12b), possibly caused by the special characteristics of the sterilization cycle seen in Figure 7. Slightly higher fit inaccuracies were found as well, but professionals at IRCSS declared that the product could still be used in procedures. The material that showed the highest fit inaccuracies was nylon, as seen in Figure 13, in which a clear deformation on the flat surface of contact with the cutting blade and total deformation of the blade guide holes made the part useless for the proposed application.

### 3.2. Autoclave Temperature Analysis Results—Sterilization Test

**Figure 14:**
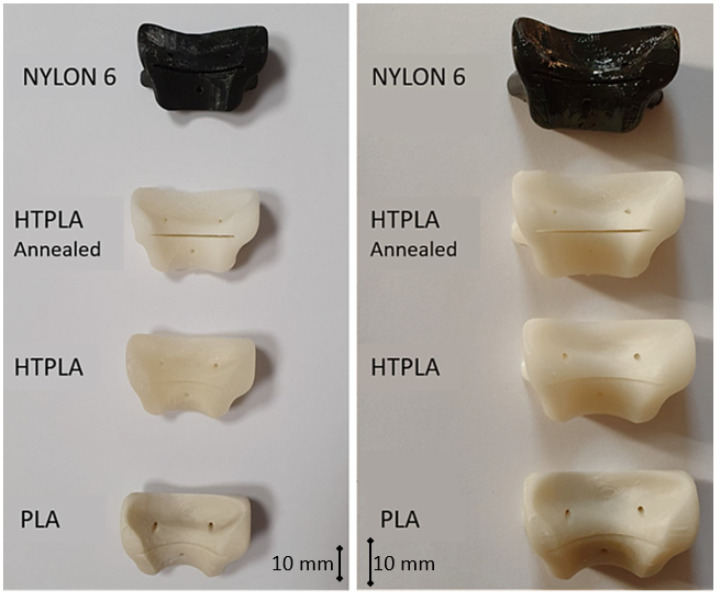
Summary: comparative view of tested masks before and after sterilization process.

Each material behaved differently after the sterilization test. Coloration was different in all materials with autoclave, as seen in Figure 9, Figure 10, Figure 11, Figure 12 and Figure 13; nylon cutting guide showed a rather significant change in surface finish, while HTPLA and PLA guides exhibited a tanned-like discoloration, as the heating process caused internal material crystallization, which was expected, as described previously by Rogers [41]. Table 6 summarizes the measurements (in mm) of the dimensions shown in Figure 8 attained for each tested part, with an absolute error percentage against the design value. It is observed that nylon, with an absolute average deviation of 2.53%, showed the greatest deformation after sterilization, while PLA (absolute average deviation of 1.35%) and annealed HTPLA (1.81%) showed the best performance in the study. It is worth noting that neat HTPLA has shown an average deformation of 2.04%, the value of which could be expected as explained by Callister [82] and detailed in Section 3.3. Further, abrupt temperature changes consequent to the part’s cooling and heating cycles during sterilization, as reported in Figure 7, caused an uneven distribution of the crystallized structure, so the product showed an undesired result. Nevertheless, it is worth noting that PLA showed the best overall result in the group, owing mainly to its slightly higher Tg and higher molecular stability at short cycles of heating and cooling, due to a lack of an internal crystallization compound, in contrast to HTPLA. A comparative view of all materials is summarized in Figure 14. Additionally, the results obtained were possible given the compact size of the element tested and the printing method using 100% polymer infill, which are discussed in the next section.

### 3.3. Material Shrinkage and Molecular Semi-Crystallization

**Figure 15:**
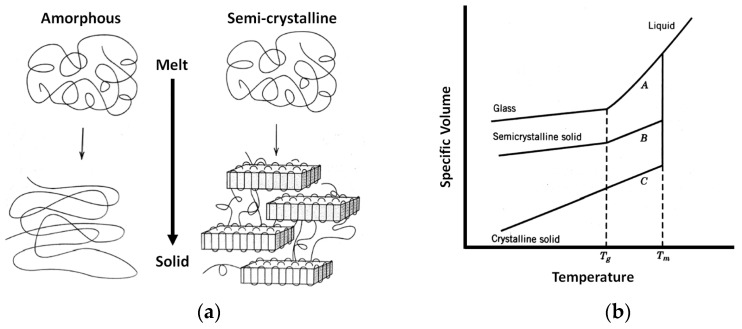
Comparative view (**a**) schematic molecular structure representation and (**b**) specific volume versus temperature, upon cooling down from amorphous (A), semicrystalline (B), and crystalline (C) polymers.

**Figure 16:**
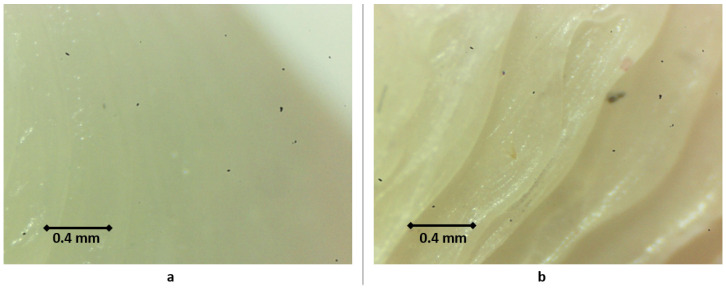
Microscopic detail of HTPLA surface: (**a**) before heat cycle and (**b**) after heat cycle.

An internal material semi-crystallization phenomenon could occur in HTPLA, as it includes a crystallization compound that, as explained by Callister [82], would cause material shrinkage due to molecular rearrangement from amorphous to a semi-crystalline (Figure 15); the behavior shown in Figure 15b will depend on the rate of cooling and heating. These phenomena do not change the microscopic structure of the piece but are consequents of molecular rearrangement that manifest as a discoloration effect on the outer side of the material. Visual changes in the material could be seen under the microscope, as shown in Figure 16. This research included both neat HTPLA and annealed HTPLA, in which its crystallizing agent, already activated in a controlled first heat cycle, would make the material resistant to later heat cycles. Finally, crystallized polymers tend to sustain higher heat cycles, as polymeric chain mobility is constrained with crystallization; therefore, the glassy state becomes steady and difficult to change at a lower temperature, thus raising the glass transition temperature, as explained by Balani et al. [83].

## 4. Discussion

The heat cycles performed in this study demonstrated the thermal inertia in polymeric materials, as described by Zhang et al. [84], which occurs as the result of a material’s thermal conductivity, as well as its density and specific heat capacity that would determine the time in which the surface temperature would reach ignition temperature once in contact with a heat interface. This phenomenon would explain why some materials are able to sustain short heating and cooling cycles at higher temperatures than their Tg without experiencing significant deformations. Thus, the PLA and HTPLA showed to be valid and functional materials for this purpose: these printing filaments are widely available, have good mechanical characteristics, and their resistance to the autoclave procedure is worth noting. The rearrangement of the polymer chains that occurred in HTPLA due to crystallization led to shrinkage stresses that resulted in additional deformation of the component. In the case of PLA, on the other hand, there is no recrystallization process, and deformations post-annealing could be linked to a relaxation of stresses due to the 3D printing process. Since the overall deformations of HTPLA are larger than those of PLA (Table 6), it is reasonable to assume that HTPLA has undergone a rearrangement process of the polymer chains. The most significant advantage found is that the annealed cutting guide in HTPLA achieved the best result of all tested specimens, as its deformation was the lowest.

Nevertheless, the results obtained indicated quick heating and cooling cycles due to the sterilization procedure shown in Figure 7, during which the items were not touched or subject to any external influence at high temperatures, therefore leading to low overall percentages of deformation. Additionally, it is worth noting that the main use of the tested objects remains at ambient temperature, and a different approach for choosing the right material would have to be applied otherwise. HTPLA was shown to be capable of sustaining repeated heat cycles if a controlled material crystallization procedure would be performed beforehand.

The obtained results contribute to the findings in the literature—namely, that 3D-printed modeling simplifies the analysis of the patient anatomy, enabling the inclusion of extra tooling, which shows the potential of this technology in health care and may help enhance the quality of preoperative planning [85]. The application of this technology in orthodontics is rapidly increasing, but the differences between the additive processes used for printing need to be further explored [86]. 

Furthermore, this technology could result in significant time savings for diverse industries. The medical sector can take advantage of this as an acceptable tool that can help in the improvement of adequate preoperative planning and training, as well as help in creating customized tooling for procedures, allowing practitioners to have reliable operations with lower impact. Procedural steps could be assessed by all parties involved and undergo a series of trials several times before the actual surgery. Furthermore, using the diagnostic methodology of CASS, the assessment could be extended to different cases and verify the effectiveness of this methodology in identifying any criticalities derived from these pathologies in the orthopedics field.

## 5. Conclusions

A regular hospital steam heat sterilization cycle by means of an autoclave was shown to not have a significant effect on the dimensional functionality for tested surgical guides printed with an FDM procedure with polymeric materials of PLA and HTPLA. However, the ideal behavior for this procedure was achieved with previously heat-treated HTPLA; this proved that an accurate annealing procedure must be performed beforehand by controlling the heating and cooling times to reach the best results in achieving material stability at high temperatures. These values would vary on the filament characteristics and provider. Additionally, PLA filament has shown results above expectations, which allows it to be considered as a valid option, in particular, due to its significantly lower costs, and also because it could be an alternative for disposable medical tooling in which a single heat cycle is needed. Furthermore, activating the crystallization compound present in HTPLA by means of previous heat treatment was shown to improve the desired performance of this polymer, turning this rather-new filament type into an ideal alternative for reusable tooling, as it sustains repeated heat cycles. On the other hand, performance defects found in the tested product using nylon suggested that this material did not meet the desired performance and might still need further research to become a valid option for FDM-obtained elements for medical purposes, and it must be stable on time and at ambient conditions. Finally, the results demonstrated how productive the engaging collaboration of doctors and engineers could be as the CASS methodology for preoperative planning proved to have high effectiveness regarding the successful creation of tooling that could be used in actual procedures with high efficacy. Finally, new material technologies with FDM filaments made of already known biocompatible polymers would allow this technology to be successfully used in operating rooms. Further research is required to analyze accurately the changes in material strength consequent to the heat cycles sustained with the chosen materials for this study for engineering purposes.

## Figures and Tables

**Table 1:** Tested materials’ suppliers with their claimed mechanical properties.

Type	Item	PLA	HTPLA	NYLON d = dry; m = moist	Based on
Filament	Diameter	1.75 mm	1.75 mm	1.75 mm	-
	Specific Gravity	1.24 (g/cc)	1.25 (g/cc)	1.12 (g/cc)	(ASTM D1505)
	Supplier	FiloAlfa^®^	Fabbrix^®^	Polymaker^®^	-
	Part Number	1PLA10002	FHT020075	200805107	-
Mechanical	Tensile Strength	47.8 Mpa	40.8 Mpa	66.2 Mpa (d),	ISO 527, (ASTM D638)
				36.4 Mpa (m)	
	Tensile Modulus	2436 Mpa	2190 Mpa	2223 Mpa (d),	ISO 527, (ASTM D638)
				1053 Mpa (m)	
	Yield Strength	-	90.9 Mpa	97 ± 1.1 Mpa (d), 41.6 ± 11.6 Mpa (m)	ISO 178, (ASTM D790)
	Elongation at Break	4.59%	5%	-	DIN EN ISO 6892-1
	Hardness-Vickers	83 Shore D	-	-	DIN EN ISO 6507-1
	Impact Strength (v-notched Izod)	17.91 kJ/m^2^	25.31 kJ/m^2^	9.6 ± 1.4 KJ/m^2^ (d)	ISO 180 (d), ISO 179 (m), ASTM D256 (t)
				17.2 ± 1.4 KJ/m^2^ (m)	
Thermal	Melting Point	145–160 °C	165–180 °C	180 °C	(ASTM D3418)
	Glass Transition (Tg)	60 °C	55–60 °C	-	(ASTM D3418)
	Fusion Temperature (Tm)	145–160 °C	165–180 °C	190 °C	(ASTM D3418)

**Table 2:** Technical specifications of E-3D ToolChanger 3D.

Printing Technology	FDM (Fused Deposition Modeling)
Printing volume	300 mm × 200 mm × 290 mm
Layer resolution	0.05 mm
Positioning accuracy	5 μm
Extruder quantity	4
Supported materials	Polymer filaments up to 300 °C
Ambient operating temperature	5–40 °C
Operational extruder temperature	max 300 °C
Operational bed temperature	max 120 °C
Software input formats	G Code (.txt)
Connectivity	Wifi—Network Managed
Weight	20 kg

**Table 3:** Optimized printing parameters.

Parameter/Material	Extrusion	Extrusion	Layer (mm)	Infill	T (°C) Nozzle	T (°C) Bed	Printing Speed (mm/s)
	Multiplier	Width					
	(%)	(mm)					
PLA	1.03	0.42	0.15	100%	200	65	50
HTPLA	0.97	0.42	0.15	100%	220	70	50
NYLON 6	0.93	0.42	0.15	100%	250	40	50

**Table 4:** Sterilization parameters according to EN 285:2015.

Temperature (C)	Pressure (Bar)	Time (Exposure) (min)
112	0.5	30
121	1	15
134	2	10

**Table 5:** Regular autoclave process.

Step	Condition	Temperature (°C)	Pressure (kPa)	Time (min)
Conditioning	-	79.6	93.4	0:00
(5 Steps)	empty + vapor steps	78.40→116.50	varies on each cycle 91.2 (min), 182 (max)	0–21.00
Heating	-	71.9	28.6	22
		134.7	312	27
Sterilization	-	134.8	311.4	28
		134.8	311.9	32
Wash	-	134.8	311.8	32
			25	
Addition	-	20	-	34
Drying	-	89.8	86.7	55
			25	
Drying	-	96.8	24.8	58
Ventilation	-	94	17.4	60
End	-	89.2	98.6	61

**Table 6:** Measurements results obtained after sterilization process.

**D1 (mm)**	**CREO**	**Not Sterilized**	**PLA**	**HTPLA**	**HTPLA-Ann**	**Nylon 6**
Media	20.00	19.90	19.46	19.44	19.03	20.4
Absolute deviation (mm)	-	0.105	0.54	0.56	0.97	0.4
Deviation %	-	0.53	2.7	2.8	4.85	−2.02
**D2 (mm)**	**CREO**	**Not Sterilized**	**PLA**	**HTPLA**	**HTPLA-Ann**	**Nylon 6**
Media	39.21	39.05	38.76	38.41	38.82	38.59
Absolute deviation (mm)	-	0.16	0.45	0.8	0.37	0.62
Deviation %	-	0.41	1.15	2.05	0.99	1.57
**D3 (mm)**	**CREO**	**Not Sterilized**	**PLA**	**HTPLA**	**HTPLA-Ann**	**Nylon 6**
Media	40.35	40.28	40.56	40.03	39.95	38.99
Absolute deviation (mm)	-	0.07	0.21	0.32	0.4	1.36
Deviation %	-	0.17	−0.52	0.79	0.98	3.37
**D4 (mm)**	**CREO**	**Not Sterilized**	**PLA**	**HTPLA**	**HTPLA-Ann**	**Nylon 6**
Media	24.55	24.42	24.3	23.93	24.44	23.9
Absolute deviation (mm)	-	0.13	0.25	0.62	0.11	0.65
Deviation %	-	0.53	1.03	2.53	0.43	2.65

## Data Availability

The data presented in this study are available on request from the corresponding author.
